# Discoverability and characteristics of health and fitness apps for deaf/hard-of-hearing and blind/low-vision users

**DOI:** 10.3389/fdgth.2026.1864883

**Published:** 2026-08-17

**Authors:** Sarah M. Hall, Becca Greenhalgh, Kayla Litster, Tasha Anderson, Savannah Rencher, Hannah Mitchell

**Affiliations:** Department of Public Health, Brigham Young University, Provo, UT, United States

**Keywords:** blindness, deafness, exercise, health status disparities, health promotion, mobile applications, persons with disabilities

## Abstract

**Purpose:**

Regular physical activity improves health, yet Deaf/hard-of-hearing (DHH) and blind/low-vision (BLV) individuals face persistent barriers to participation. Accessible fitness apps could improve health equity if they are discoverable in mainstream marketplaces and include meaningful accessibility features that support participation.

**Materials and methods:**

A scoping search of the Apple App Store was conducted using nine DHH/BLV-related terms. After consolidating 3,265 search returns and removing duplicates, 1,735 unique apps were analyzed. Apps were classified by DHH/BLV relevance using a mixed inductive–deductive content analysis, and features and functions that support health or physical activity were consolidated. DHH/BLV-tailored fitness apps were then directly tested using a predefined accessibility assessment framework informed by the Web Content Accessibility Guidelines (WCAG).

**Results:**

Most returned apps (59.9%) were general health/fitness apps or unrelated. Only 15.7% were DHH-relevant while 6.6% were BLV-relevant, with an additional 0.5% overlapping across both groups. Most DHH-relevant apps focused on hearing-aid management, environmental or digital accessibility, or sound amplification. Most BLV-relevant apps focused on environmental accessibility, provider tools, therapeutics, or rehabilitation. Only four fitness apps tailored to DHH/BLV users were identified across the full corpus. These apps appeared inconsistently across search terms and were sometimes ranked low in search results, indicating substantial discoverability barriers. Accessibility features varied, and several apps had limited offerings or access barriers, including subscription costs and external hardware requirements.

**Conclusion:**

Disability-tailored fitness apps for DHH and BLV users were rare in Apple App Store search returns and were not consistently discoverable through relevant search terms. Improving access to these tools will require better marketplace metadata, accessibility labeling, ranking practices, and filtering options so users can more easily identify apps that match their needs. DHH/BLV community testing is needed to ensure fitness tools are accessible, usable, affordable, and effective.

## 1 Introduction

### 1.1 Disability and health

Approximately fourteen percent of the world’s adult population lives with disability (1). Despite equity-oriented legislation, substantial health disparities persist for persons with disabilities (PWD), including Deaf or hard-of-hearing (DHH) and blind or low-vision (BLV) individuals. PWD are more likely than their non-disabled peers to experience secondary physical and mental health conditions such as obesity, type 2 diabetes, cardiovascular disease, stroke, pain, fatigue, fall-related injury, cancer, and depression (2–4).

Health disparities are shaped by biological, social, and structural determinants, including transportation and housing barriers, limited social and organizational support, inaccessible health and wellness programs, marginalization, and inequities in education, employment, and socioeconomic resources (5, 6). Physical activity barriers may include psychological factors (e.g., low self-esteem, negative body image, injury anxiety), resource constraints (e.g., transportation, fitness membership costs), and environmental inaccessibility (e.g., lack of accessible facilities and equipment) (7, 8). Nutrition barriers include low nutrition knowledge and lower fruit and vegetable intake among Deaf adults (9), complications in portioning and calorie tracking for those who are legally blind (10), and functional limitations that impede meal preparation and grocery shopping (11). Together, these barriers suggest a need for accessible tools that support physical activity and nutrition-related behaviors.

### 1.2 Mobile fitness applications and disability

Accessible mobile fitness technologies offer a scalable approach to reducing health and fitness disparities for DHH/BLV communities. Regular physical activity can mitigate or slow the progression of secondary conditions (12) and confer anti-inflammatory and immune benefits (7). For BLV and deafblind users, tailored exercise programs can reduce balance, posture, and coordination challenges and improve strength, mobility, and spatial awareness essential for independent navigation (13). For DHH users, accessible programs that minimize auditory-only cues through captions, images, clear visual demonstrations, sign-language content, and haptic timing prompts can decrease barriers and support sustained engagement (14–17).

Mobile technologies have the potential to support participation in fitness activities. However, their public health value depends in part on whether intended users can find them. Discoverability is defined in this study as the degree to which intended users can locate and recognize relevant tailored apps using reasonable keyword searches and browsing strategies in mainstream marketplaces. Little is known about what DHH/BLV users encounter when searching mainstream app marketplaces for relevant health and fitness tools. This gap is important because app stores are not fixed catalogs; they are ranked and searchable interfaces that shape whether relevant tools are visible, recognizable, and practically accessible. Existing evaluations of disability-specific mobile fitness tools often emphasize usability in populations with mobility disabilities (8, 18, 19) or neurological conditions (20). Less is known about whether fitness apps specifically tailored to DHH or BLV users are discoverable and what adjacent health-related app types appear in these searches.

### 1.3 Study aims

To address these gaps, the present study investigates (1) the marketplace discoverability of DHH/BLV-tailored fitness apps, (2) the characteristics of adjacent DHH/BLV-targeted health apps, and (3) the observable features and potential access barriers of existing fitness apps tailored to DHH/BLV users. First, we conducted a scoping App Store search to characterize the app ecosystem reflected in keyword search returns, including the proportion of apps truly tailored to DHH/BLV users compared with unrelated, general-audience, or general hearing/vision-related apps. Second, we used qualitative content analysis to document the main functions and features of health-related apps explicitly designed for DHH and BLV users. Third, we assessed the subset of DHH/BLV-targeted fitness apps to identify observable accessibility features, available activities, and potential barriers to use.

## 2 Materials and methods

### 2.1 Search strategy and corpus creation

The research progressed in four phases. Phase 1 focused on developing and executing the App Store search strategy, compiling results, and removing duplicates to create the analytic corpus. Phase 2 focused on developing and applying a codebook to classify apps into categories within the corpus. Phase 3 focused on synthesizing DHH- and BLV-targeted health-related apps, including compilation of subcategory features. Phase 4 focused on an accessibility assessment of DHH and BLV-tailored fitness apps.

During Phase 1, the researchers conducted a scoping search in the Apple App Store during late 2025. Apple devices are widely used among BLV screen-reader users, particularly in North America, supporting the relevance of the Apple App Store as an important marketplace for investigating BLV app discoverability. This may in part reflect the availability of built-in accessibility features such as VoiceOver, Magnifier, braille display support, and other accommodations. Only one marketplace was analyzed to provide a controlled and standardized search environment using consistent devices, operating systems, and search procedures. This approach reduced variability from cross-platform differences in app availability, metadata, and ranking algorithms. Since the study’s aim was to characterize what DHH/BLV users might encounter within a mainstream marketplace search environment, this approach allowed for an in-depth assessment of one major app ecosystem rather than a partial comparison across multiple stores.

All searches were performed on iPads running the most recent version of iPadOS available at the time of data collection and were conducted using the App Store search interface, with results captured as returned by Apple’s ranking algorithm at the time of each query. Prior search personalization was minimized by conducting searches on factory-reset iPads running the same version of iPadOS. All results returned by the App Store search function at the time of each query were included in the corpus.

To operationalize the search for health and fitness, we used a two-step retrieval approach. First, disability-related terms were queried within the App Store’s Health & Fitness category. Second, each search was repeated in the general App Store using disability-related terms paired with the terms *health* and *fitness*. Both search locations were used to reflect plausible user search strategies: browsing within a health-related category and searching the broader marketplace. Category-limited searching captured apps indexed by Apple as Health & Fitness, whereas general App Store searches captured relevant apps that may support health, fitness, rehabilitation, accessibility, or participation but were classified under another marketplace category. This approach also helped identify tailored fitness apps that may not have been categorized by developers within Health & Fitness.

In accordance with American Psychological Association (APA) (21) guidance on bias-free disability language and the National Association of the Deaf (NAD) (22) recommendations on community-preferred terminology, the search strategy prioritized identity and community-aligned keywords. The nine search terms were selected to balance community-preferred terminology, commonly used clinical or marketplace terminology, and terms likely to be used by consumers when searching for DHH/BLV-related tools. For DHH-related searches, *Deaf* and *hard of hearing* were included as community-aligned identity terms. *Hearing loss* and *hearing impaired* were also included because these terms remain common in app descriptions, clinical language, and mainstream metadata and queries (23). These terms were used for retrieval purposes only; their inclusion does not imply endorsement but instead reflects the practical need to capture apps that may be labeled with non-preferred terminology despite being relevant to Deaf and hard-of-hearing users. For BLV-related searches, *blind*, *low vision*, *visual impairment*, and *legal blindness* were included to capture both identity-based and clinical/functional terminology. *DeafBlind* was included to capture apps that may explicitly target users with combined hearing and vision disabilities.

The nine key terms were individually queried. The initial searches were limited to the App Store’s Health & Fitness category. Counts reflect the number of results returned by the store’s search function at the time of query: *Deaf* (*N* = 126), *hard of hearing* (*N* = 178), *DeafBlind* (*N* = 176), *hearing loss* (*N* = 170), *hearing impaired* (*N* = 163), *blind* (*N* = 158), *visual impairment* (*N* = 168), *low vision* (*N* = 207), and *legal blindness* (*N* = 185). To broaden capture beyond category-limited indexing, each search was repeated in the general App Store, pairing the prior disability-related terms with the keywords *health* and *fitness*. This yielded: *Deaf* (*N* = 176), *hard of hearing* (*N* = 180), *DeafBlind* (*N* = 210), *hearing loss* (*N* = 170), *hearing impaired* (*N* = 193), *blind* (*N* = 171), *visual impairment* (*N* = 212), *low vision* (*N* = 222), and *legal blindness* (*N* = 200). Searches were not repeated at multiple time points and thus reflect a single-point analysis of discoverability at the time of the search.

All search returns were consolidated into a single corpus (*N* = 3,265). Duplicate removal was conducted in sequential steps. First, all 3,265 search returns were compiled into a spreadsheet and sorted alphabetically by app title. Second, exact duplicate titles were removed. Third, the alphabetized list was manually reviewed to identify residual duplicates not captured by exact title matching, including localized or rebranded listings and edition variants such as Pro, Plus, Lite, HD, Premium, Trial, or Demo versions. Fourth, apps without an English App Store listing description were excluded. This process yielded a final analytic corpus of 1,735 unique apps. Since app store inventories and rankings change over time, the corpus reflects a time-bound snapshot of Apple App Store search returns at the time of data collection rather than a stable or permanent representation of app availability or ranking.

### 2.2 Codebook development and app classification

Phase 2 of the analysis consisted of categorizing the corpus of unique apps. The research team used a mixed inductive–deductive content analysis (24). First, one researcher used an open-coding procedure to conduct an initial grouping of apps based on their titles and App Store descriptions, consistent with established approaches for inductive qualitative coding and codebook development. This preliminary review resulted in five overarching categories: (1) apps explicitly designed for or marketed to DHH populations; (2) hearing-related apps that did not specifically target DHH users, such as general hearing screenings, sign language learning, tinnitus-related apps, generic hearing health apps; (3) apps explicitly designed for or marketed to BLV users; (4) vision-related apps that did not specifically target BLV users, such as vision screenings, general eye health, colorblind tests; and (5) other, such as apps intended for the general population with no explicit focus on hearing or vision, or other unrelated offerings.

Before full coding began, additional coders were oriented to the study aims, reviewed the initial category definitions, and practiced applying codes to a shared subset of apps. The team discussed coding decisions during this training phase to refine definitions, clarify boundary cases, and promote consistent application of the codebook. Four researchers independently reviewed a subset of the corpus (approximately 200 apps) to inductively develop an initial codebook that defined subcategories within each overarching category based on app elements including functional purpose, the primary user need addressed, or assistive feature type. After independent review, the research team met to compare individually generated subcategory labels, refine subcategory definitions, resolve ambiguities, and consolidate the codebook for consistent application across coders.

When coders encountered apps that did not align with existing categories, subcategory definitions were updated, expanded, or refined to incorporate new functions or feature types. When needed, new subcategories were created with rationales grounded in the apps’ content and intended audience. Through this process, the initial *accessibility* subcategory was divided into *digital accessibility* and *environmental accessibility*. *Digital accessibility* described using the device to enable or improve access to content and tasks on the device itself, such as live captions for phone calls, device media captions, and mobile braille keyboards. *Environmental accessibility* described apps that use the device to help users access or interact with the outside world, such as object identification, physical navigation, and live captions for conversations outside the device. *Provider/insurer connection* was separated from *provider tools*. *Provider tools* were defined as mobile tools to help providers serve patients, such as patient information tracking, telehealth visits, and screenings. *Provider/insurer connection* was defined as tools to connect patients to providers, insurance companies, or retail stores. *Hearing aid* apps and *sound amplifier* apps were divided into two distinct categories. The *vision test/screening* subcategory was redefined to include color blindness tests and screenings; the *colorblind support* category was redefined as tools to assist individuals with navigating colorblindness. New categories added through this process included *BLV education, DHH fitness club, hearing simulator, DHH community/religion,* and *BLV games*. Games that were primarily therapeutic in nature were retained in the therapeutic/rehabilitation category.

The finalized codebook was applied to the full dataset using a deductive coding process (see Supplementary Material). When an app could plausibly fit into more than one subcategory, it was assigned to one category only based on its primary function as described in the App Store listing. As an exception, apps explicitly designed for both DHH and BLV users were retained in both the DHH-specific and BLV-specific categories. Three researchers independently coded each app into a relevant subcategory based on codebook category definitions and app descriptions. Inter-rater reliability across the 1,735 unique apps was excellent for pre-consensus subcategory assignments (Fleiss’ *κ*=0.88). All differences in assigned subcategories across coders were flagged as disagreements requiring review. The coders convened to review all discrepancies, defined as any app for which at least one coder assigned a different subcategory than the others, and to reach consensus through discussion. A fourth researcher reviewed the final category assignments and app descriptions for all apps flagged for initial discrepancies. Final codes were assigned only after consensus was reached.

### 2.3 Synthesis of DHH/BLV-targeted apps

Phase 3 focused on extracting descriptive information from App Store listings for DHH- and BLV-targeted apps, describing functions and features within each subcategory, and compiling DHH- and BLV-specific apps into summary tables during early 2026. The research team initially planned to conduct a feature-focused analysis restricted to apps explicitly targeting physical fitness or nutrition for DHH/BLV users. However, the search yielded very few apps meeting this narrow criterion (*N* = 4). To more accurately characterize the app ecosystem relevant to health-related apps for DHH and BLV users, the analytic scope was broadened to include adjacent app types that could plausibly support health behaviors, healthcare engagement, and safer participation in daily activities, including accessibility tools, rehabilitation/therapy supports, communication access tools, community support, education tools, and disease management utilities. This expanded approach maintained an explicit focus on DHH and BLV relevance while capturing specific features that may support overall health and well-being.

For all apps classified in Phase 2 as DHH- or BLV-specific, the research team extracted key information from each App Store listing into a structured spreadsheet including the app name and the listing’s description. When needed, researchers also reviewed screenshots and feature summaries presented in the listing to resolve ambiguity about app function or target audience. Because app marketplaces are dynamic, 24 apps identified during Phase 1 were no longer available within the App Store at the time of Phase 3 extraction. These apps were documented as unavailable and were not included in the features and functions synthesis.

Using extracted descriptions, the team synthesized common and unique features and functions available across apps within each subcategory. Apps that aligned closely with the dominant feature offerings were summarized within their respective clusters, whereas apps with uncommon or novel functions were described individually to preserve the breadth of features and functions represented in the dataset. The refined groupings were used to generate summary tables.

### 2.4 Accessibility assessment of fitness-focused apps

During Phase 4, researchers downloaded and directly tested each app classified as DHH- or BLV-targeted fitness or nutrition to verify functionality beyond the listing description. Four fitness apps were found, whereas no nutrition-related apps targeting DHH or BLV users were found. Phase 4 included gathering information about the developer, direct and indirect user costs, fitness app offerings such as online workout availability, livestream workouts, fitness tracking, goal setting and progress, networking and interaction, reminders and scheduling, and accessibility features.

Accessibility feature criteria were informed by the World Wide Web Consortium’s Web Content Accessibility Guidelines (W3C) (25) and applied using a predefined evaluation framework, enabling consistent assessment across testers. This assessment was designed to provide a brief overview of observable accessibility features and was not intended to establish full WCAG conformance. Three researchers independently reviewed accessibility features for BLV users, which included non-visual workout guidance, size and contrast of text, magnification and zoom, haptics and tactile cues, voice commands or control, and navigation and touch targets. The same process was repeated for DHH users for accessibility features including audio-independent instructions, captions and subtitles, searchable transcripts for workouts or classes, visuals or haptics, and modified alerts or notifications. Since no DHH or BLV participants were involved in this phase, the assessment should be interpreted as an evaluation of the presence or absence of easily observable accessibility features rather than actual accessibility or lived experience of the intended users.

## 3 Results

### 3.1 Corpus composition

**Figure 1:**
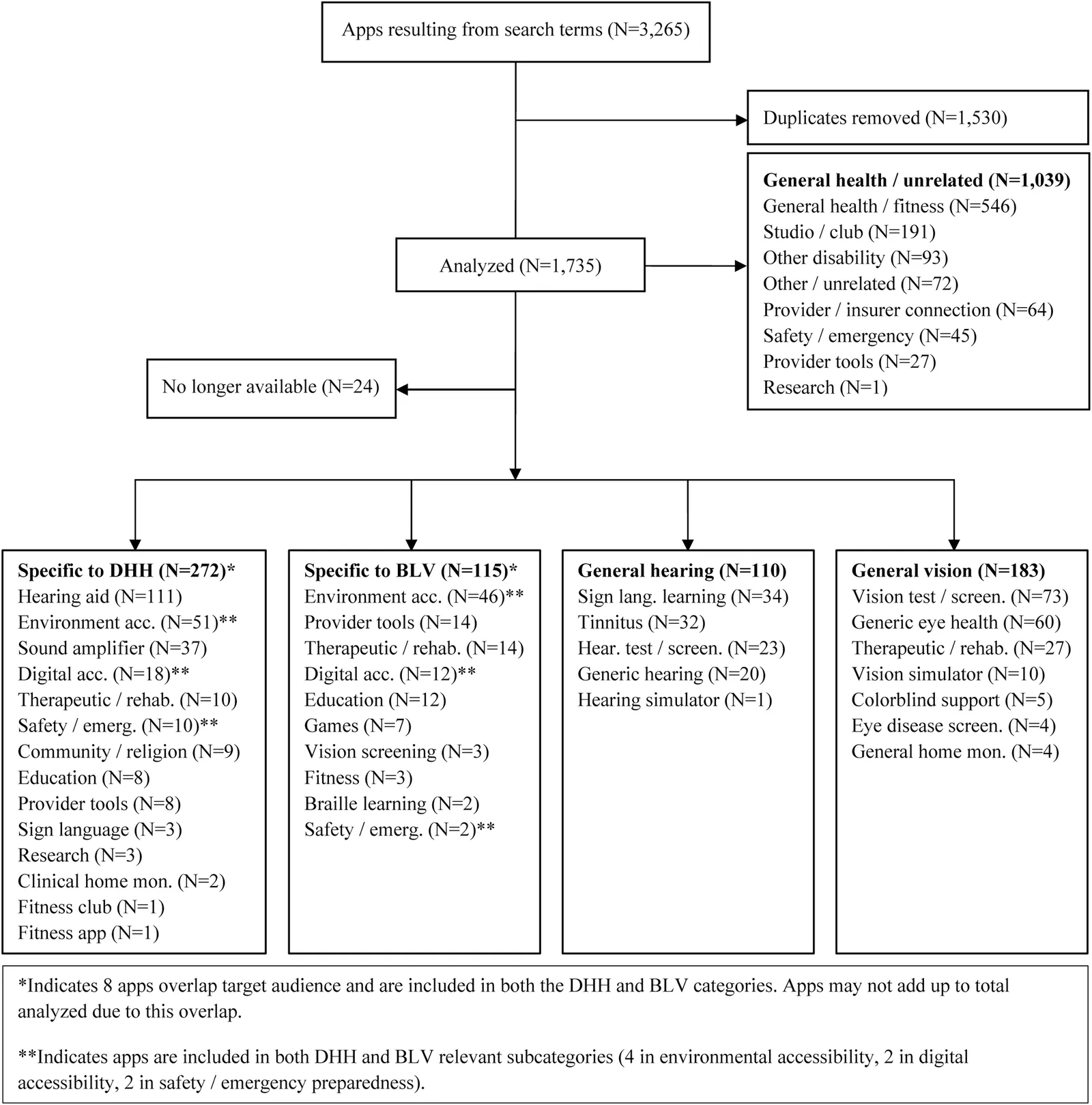
Flow diagram of search results.

**Figure 2:**
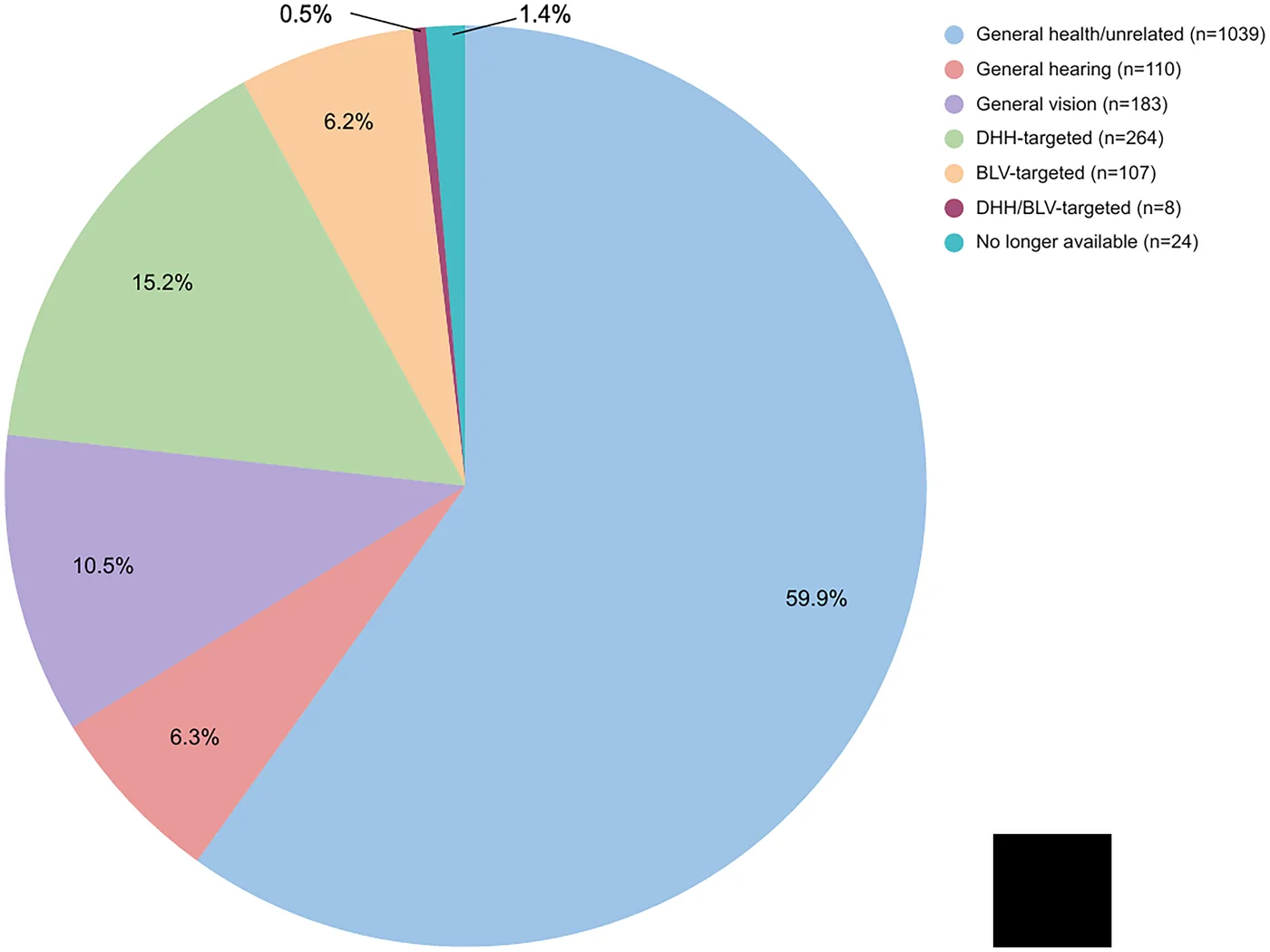
Full corpus categorization.

Figure 1 provides a flowchart of app categorization for the full analyzed corpus, and Figure 2 shows the proportional breakdown of the full analyzed corpus. 24 apps were no longer available at the time of categorization. Among the final corpus of unique apps (*N* = 1,735), the majority (59.9%, *N* = 1,039) were apps pertaining to general health or fitness but not specific to hearing or vision or were unrelated. These included general health and fitness apps (*N* = 546), studio and club fitness apps (*N* = 191), other disability apps not related to DHH or BLV populations (*N* = 93), apps unrelated to health or fitness (*N* = 72), provider/insurer/retail connection apps (*N* = 64), safety/preparedness apps (*N* = 45), provider tool apps (*N* = 27), and research apps (*N* = 1). Additionally, 6.3% (*N* = 110) pertained to general hearing health (not DHH-targeted) and 10.5% (*N* = 183) pertained to general vision health (not BLV-targeted). Further, 15.7% (*N* = 272) were relevant to DHH populations, and 6.6% (*N* = 115) were relevant to BLV populations, with 8 of these apps (0.5%) overlapping across the two groups**.** In total, 379 apps were relevant to DHH and/or BLV users, but only four were identified as DHH/BLV-tailored fitness apps. No DHH/BLV-tailored nutrition apps were identified. The DHH- and BLV-relevant app results are presented separately from the fitness-specific findings to distinguish the broader disability-relevant app ecosystem from the much smaller set of apps that directly provided tailored fitness content.

#### 3.1.1 General hearing apps

General hearing subcategories included sign language learning (*N* = 34), tinnitus therapeutic apps (*N* = 32), hearing testing or screening (*N* = 23), generic hearing health (*N* = 20), and a hearing simulator (*N* = 1). Sign language learning apps were defined as apps that taught any type of sign language, catered to the general public, and were not intended for DHH individuals. Tinnitus therapeutic apps were those whose primary function was to provide therapeutic relief for individuals experiencing tinnitus. Hearing testing or screening apps included those designed for the general public to assess hearing ability. Apps that offered hearing tests or screening services but were provider-exclusive, connected to hearing aid configuration, or linked to a specific hearing provider were categorized instead as provider tools, hearing aid-related apps, or provider/insurer connection apps. Generic hearing health apps included those that aimed to improve hearing, measure real-time noise levels, customize audiograms to personalize hearing profiles, and provide educational resources on hearing protection. One app was categorized separately as a hearing simulator because it was created for non-DHH individuals to experience what it is like to be deaf or hard-of-hearing.

#### 3.1.2 General vision apps

General vision subcategories included vision testing or screening (*N* = 73), generic eye health (*N* = 60), therapeutic or rehabilitation apps (*N* = 27), vision simulators (*N* = 10), colorblind support (*N* = 5), eye disease screening (*N* = 4), and general clinical home monitoring (*N* = 4). Vision testing/screening apps included those that offered at-home visual acuity examinations to detect color blindness, astigmatism, and contrast clarity. Features of generic eye health apps included eye exercises intended to maintain or improve eyesight, reminders for users to take screen time breaks and blink, eye health tips and education, pupil distance measurement for personalizing eyeglass frames, and apps that screen for or provide support for visual stress symptoms. Therapeutic or rehabilitation apps included those that aim to assist in sight recovery and treat or relieve symptoms from vision disorders or ocular diseases. Vision simulator apps offered a BLV simulation experience for sighted individuals. Colorblind support apps provided resources for colorblind individuals, including color recognition and tools to help users distinguish colors. Apps that aid in monitoring general eye health and connecting users with an eye care professional were categorized as general clinical home monitoring.

### 3.2 Attributes of health apps specific to DHH

**Figure 3:**
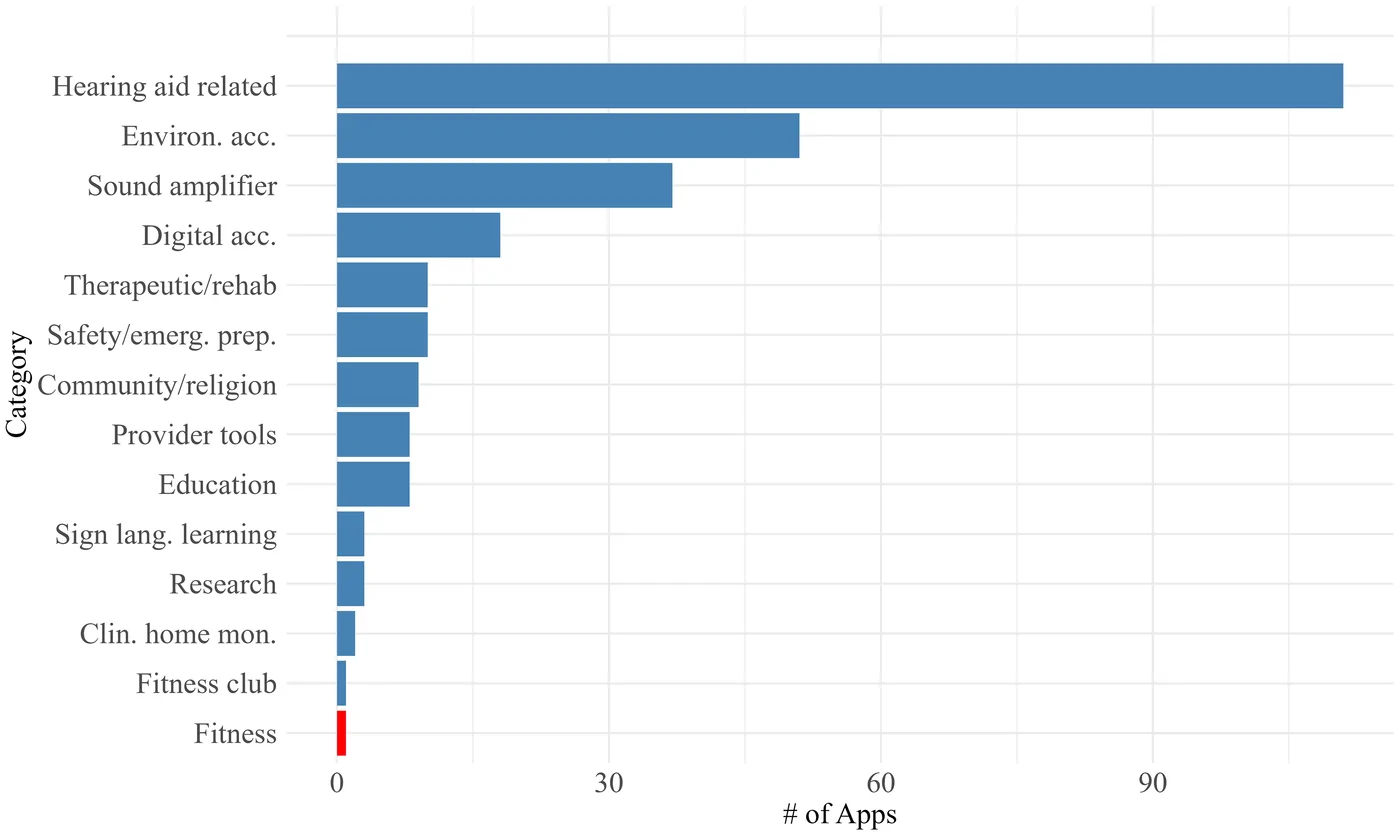
Categories of DHH-specific apps.

DHH-specific apps found were most commonly *hearing aid-related* (*N* = 111), followed by *environmental accessibility* (*N* = 51, including 4 dual-coded), *sound amplifier* (*N* = 37), *digital accessibility* (*N* = 18, including 2 dual-coded), *therapeutic/rehabilitation* (*N* = 10), *safety/emergency preparedness* (*N* = 10, including 2 dual-coded), *community/religion* (*N* = 9), *education* (*N* = 8), *provider tools* (*N* = 8), *sign language learning* (*N* = 3), *research* (*N* = 3), *clinical home monitoring* (*N* = 2), *fitness club* (*N* = 1), and *fitness* (*N* = 1). The dual-coded apps applied to both DHH and BLV users and were included in both categories. Table 1 presents DHH-specific apps organized by subcategory, with brief descriptions of common functions and features alongside examples of selected apps within each subcategory. Figure 3 visualizes the distribution of DHH-specific apps across subcategories.

**Table 1:** Apps targeting Deaf and hard-of-hearing populations

Category (N)	Description	App Examples
Hearing Aid Management and Control (N = 111)	Allows users to control hearing aids from mobile device (e.g., adjust volume, frequency, noise focus, monitor battery)	RxEars, GN Hearing Tuner, Widex Moment, Ears G29
	Personalize listening experience (e.g., custom sound profiles, listening controls, geo-tagging)	TruLink Hearing Control, Sonify Hearing, Ears G29
	Use a mobile device to find lost hearing aids	Beltone HearPlus, Find My Hearing Aids App
	Update hearing aid firmware	LIZN Headpieces
	Conduct fittings	Hearing Adaptation
	How-to videos and guides for caring for hearing aids and connecting to device	AGXR Attune, Tuned Hearing, My SimpleSound
	Track hearing progress	KINDconnect
Environmental Accessibility (N = 51)^a^	Real-time captions for live conversations	Live Transcribe, Wushi Live Transcribe Voice
	Communicates loud noises through a customizable alert tool using vibrations, LED flashes, and notifications to phone or watch	BeAware Deaf Assistant
	Connects users to video interpreters for in-person conversations	Deaftawk, App MyGroup
	Uses flashing lights, vibrations, and sounds to wake DHH users	Deaf Wake
Sound Amplifier (N = 37)	Amplifies sound through calibrated microphones and receivers	Clear AMP, Hear Me Well, Super Ear Audio Enhancer
	Amplifies the sounds of pre-recorded audio clips	Super Hearing Clear Sound AMP
	Reduces ambient noise to help the user hear relevant nearby conversations	able EQ Hearing Aid, HearLive: Audio Enhancer
	Amplifies live sound and provides a live text transcription	Hearing Aid by Shesek
Digital Accessibility (N = 18)^a^	Live captions to assist DHH individuals in making and receiving phone calls	HearClear, CaptionCall
	Phone service for DHH individuals	T-Mobile IP Relay
	Audiovisual books displaying voice, text, and face for lipreading training	Clearly Audiobooks
	Interpreter services for phone calls	Convo VRS, Z5 Mobile
	Audio descriptions and closed captioning for movies and shows viewed on device	Spectrum Access: Enabled Media
	Transfers voice calls to video calls	Sorenson Wavello, Sivo
Therapeutic / Rehabilitation (N = 10)	Supports hearing rehabilitation for users with cochlear implants, hearing aids, and aphasia	Hear Glue Ear, HearApp, Auribus
	Rehabilitation exercises for users of hearing technology	ReDi, AudioFitness, WordSuccess
	Supports children adapting to new communication styles after hearing loss	HearApp Kids, BabyBeats Resource
Safety / Emergency (N = 10)^a^	Allows deaf and/or deafblind individuals to call for emergency, fire department, or police services	WIS Emergency for deaf people, accessSOS: 24/7 Emergency Help
	Supports emergency video calls and sign language reporting	112 Georgia, Deaf JO 114
	Gives first responders quick access to disability-related information	AdaptCare
	Provides emergency preparedness toll and visual communication resources	Emergency PreparednessToolkit
	Helps users share their location with emergency services	Sosfy Help, accessSOS: 24/7 Emergency Help
	Supports safety in ride-sharing and social situations with preset messages	Text to 911
Community / Religion (N = 9)	Provides parenting and family relationship resources	Hands & Voices
	Inspirational content tailored towards overcoming barriers for DHH individuals	No Barriers with Joel Barish
	Offers news, social networking, and communications services focused on d/Deaf, hard-of-hearing, and Children of Deaf Adults (CODA)	Tive
	DHH-targeted platform for individuals to access and share scriptural content	Deaf Missions Video
	Locate Deaf churches and stay connected with these communities	Deaf Church Where
Education (N = 8)	Strategies and tips for coping with hearing loss, including cochlear implantation	5 Keys Communication, Cochlear Copilot
	Communication tips that users can share with family and friends	Cochlear Copilot
	Helps parents navigate newborn hearing screening and explain hearing loss to children	Hear & Watch, Phonak Leo, & Maine’s EHDI Process
	Vocational training apps available in sign language	Sign Language App- ICT
Provider Tools (N = 8)	Conduct hearing screenings and fit personal audio amplifiers and hearing aids	Hearing Test By Audicus, EarScript, Audicus THT
	Provides a space for DHH providers to share information and collaborate	ASHA Community
	Helps providers identify people who may need hearing services	SHOEBOX QuickTest
	Patient appointments and ear imaging	Heardat
Sign Language Learning (N = 3)	Teaches ASL to Deaf students, their families, and communities	ASLdeafined
	Sign language courses meant to teach marketable skills to DHH individuals	SignMedium
	Pakistan sign language resources for deaf students and their families	PSL by Deaf Reach
Research (N = 3)	Hearing aid research	MyHearingExperience
	Users and researchers can find or create projects dedicated to accessibility research	Accessibility research
	Apple conducts research on a variety of topics including disabilities	Apple Research
Clinical Home Monitoring (N = 2)	Patients share smartphone hearing test results with their provider	Meludia Hearing Better
	Provides patient monitoring before and after cochlear implant surgery	CIQOL
Fitness Club (N = 1)	Shares information about sports opportunities for DHH individuals in Kuwait	Kuwait Sports Club for Deaf
Fitness (N = 1)	Uses vibration and flashing lights to promote freestyle dancing	BW Dance App for Deaf and HOH

a Indicates apps are included in both DHH and BLV relevant subcategories (4 in environmental accessibility, 2 in digital accessibility, 2 in safety/emergency).

#### 3.2.1 Hearing-aid related

Hearing aid-related apps (*N* = 111) were designed to support the use, customization, and maintenance of hearing aid devices. Device integration and control features included monitoring battery life, adjusting volume and frequency settings, switching between listening programs, locating misplaced devices, and installing firmware updates. Audio streaming capabilities enabled direct transmission of phone calls, music, and other media from mobile devices to hearing aids. Personalization tools included advanced sound environment adjustments and adaptive listening modes. Some apps also incorporated data tracking, allowing users to monitor usage patterns and listening environments over time. Most apps were developed for specific hearing aid product lines and could not be used across devices from different manufacturers.

#### 3.2.2 Environmental accessibility

Environmental accessibility apps (*N* = 51) contained features supporting DHH users’ interaction with and awareness of their physical surroundings. Communication access tools included live captioning and on-demand video remote interpreting (VRI) to facilitate in-person conversations. These tools functioned as real-time interpretation systems, enabling users to convert spoken language into text or sign language during face-to-face interactions. Alerting and notification systems included vibration-based alarms, flashing light signals, and amplified sound alerts for events such as doorbells, timers, and emergency warnings. Some apps also integrated with external smart devices, such as door sensors or home alert systems, to extend accessibility within the home environment. These features allowed DHH users to utilize mobile devices as tools for both communication and environmental awareness.

#### 3.2.3 Sound amplifier

Sound amplifier apps (*N* = 37) focused on enhancing auditory input from the user’s immediate environment. Audio amplification features included microphone-based sound boosting, ambient noise enhancement, and directional listening modes that prioritize sounds from specific sources. Speech clarity tools included background noise reduction, frequency filtering, and voice enhancement to make speech easier to understand in noisy settings. Additional customization options often included adjustable amplification levels, frequency-specific sound controls, and preset settings for different listening environments such as conversations, lectures, noisy rooms, or outdoor settings. Some apps incorporated real-time transcription or captioning alongside amplified audio, allowing users to both hear and read spoken content.

#### 3.2.4 Digital accessibility

Digital accessibility apps (*N* = 18) promoted access to communication, media, and other content through digital devices. Communication support features included live captioning for phone calls, voicemail transcription, and sign language interpretation services integrated into calling platforms. Media accessibility tools included closed captioning or sign language support for streaming video, audiovisual books, or other entertainment and educational resources. Additional features included sound detection systems that use the digital device to notify users of important environmental sounds such as alarms or crying babies, accessible customer service tools designed for DHH users, and platforms that integrate multiple accessibility services into a single interface.

#### 3.2.5 Therapeutic and rehabilitation

Therapeutic or rehabilitation apps (*N* = 10) provided rehabilitation exercises for individuals with cochlear implants, hearing aids, auditory processing disorders, or language disorders such as aphasia. This included apps that aimed to reduce learning and developmental delays for children, and to practice identifying words and sounds for adults experiencing hearing loss. These exercises support DHH users in practicing auditory and speech skills while receiving feedback in real time. Common features included gamified listening activities to build familiarity with environmental sounds, resources highlighting common sound or word confusion, and personalized training plans. Four of the ten apps explicitly described features to support DHH babies and children, including using music to help babies connect to and communicate with their parents, and child-friendly word identification practice.

#### 3.2.6 Safety and emergency preparedness

Safety/emergency preparedness apps (*N* = 10) offered tools to support DHH users in emergency and high-risk situations. Emergency communication features included 911 video calling and sign language-based communication with dispatchers, reducing reliance on voice-based systems. Location-sharing tools enabled users to transmit their real-time location to first responders or trusted contacts. Preparedness resources included guidance for scenarios such as natural disasters, ride-sharing, and online meetups, with an emphasis on communication safety and situational awareness. Some apps were provider-facing, offering first responder access to patient-specific information such as disability status and communication needs to improve emergency response.

#### 3.2.7 Community and religion

Apps pertaining to community or religion (*N* = 9) supported social connection, identity, and community engagement among DHH users and hearing parents with DHH children. Social networking features enabled users to connect with others, share experiences, and participate in discussions related to Deaf culture and daily life. Connecting based on shared identity and culture was a major focus of apps within this category. Content related to travel and entertainment, specifically for the Deaf community, was also prevalent. Informational content included DHH-oriented news on topics such as health, education, careers, and parenting. Motivational and support resources provided encouragement and community-building opportunities. Spiritual and religious tools included access to scripture videos in sign language and a Deaf church locator.

#### 3.2.8 Education

Education apps (*N* = 8) provided informational and instructional resources related to hearing loss, communication strategies, and skill development. These resources were directed toward DHH individuals and others who interact with them directly. Family and caregiver resources included guidance for hearing family members on how to effectively communicate with DHH individuals. Educational media was available in several formats, often articles and videos. Parent-focused tools supported families of DHH children through educational modules, developmental guidance, and communication training. Vocational and career-oriented content, often delivered in sign language, supported job readiness and professional development. Some apps included general educational materials about hearing loss, cochlear implants, and assistive technologies. Others had specific referral materials to direct individuals to resources for care and support.

#### 3.2.9 Provider tools

Provider tool apps (*N* = 8) were designed to support healthcare professionals and specialists working with DHH patients. Clinical tools included hearing screening applications, diagnostic supports, and tools for fitting and adjusting hearing aids. These tools were often used by the provider in the office. Decision support and education features provided resources to guide clinical care and improve patient counseling. Collaboration platforms enabled providers to share knowledge, discuss cases, and stay updated on best practices. These apps aimed to increase provider knowledge related to working with DHH patients. Workflow tools included patient management systems, appointment scheduling, and communication assistance to streamline clinical operations.

#### 3.2.10 Sign language learning, research, clinical home monitoring, and fitness

Five categories contained three or fewer apps. Sign language learning apps (*N* = 3) were tailored toward sign language advancement for DHH users; two taught American Sign Language (ASL) and one provided instruction in Pakistan Sign Language. Research (*N* = 3) included one hearing aid research hub and two platforms to disseminate research relevant to DHH issues. Clinical home monitoring apps (*N* = 2) enabled patients to communicate medical results and condition monitoring information and maintain an active and ongoing relationship with their providers. The fitness club app (*N* = 1) provided information and news about DHH fitness opportunities at a Kuwait-specific club. No apps connecting users to DHH-specific fitness clubs were found for the United States. One fitness app (*N* = 1) created a freestyle dance environment through haptic and visual cues, including lights flashing in rhythm to user-uploaded songs.

### 3.3 Attributes of health apps specific to BLV

**Figure 4:**
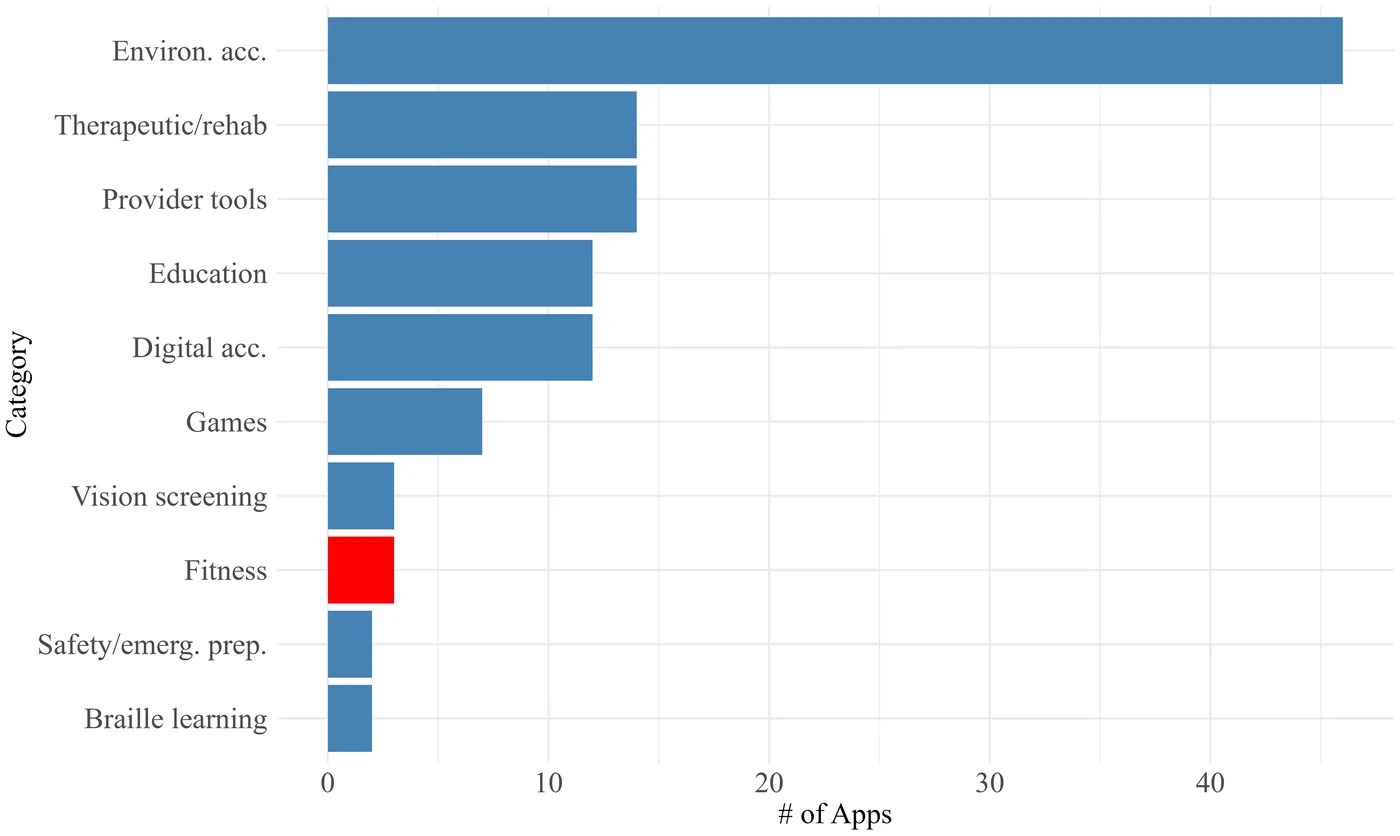
Categories of BLV-specific apps.

BLV-specific apps were most frequently classified as *environmental accessibility* (*N* = 46, including 4 dual-coded), followed by *provider tools* (*N* = 14), *therapeutic/rehabilitation* (*N* = 14), *education* (*N* = 12), *digital accessibility* (*N* = 12, including 2 dual-coded), *games* (*N* = 7), *vision screening* (*N* = 3), *fitness* (*N* = 3), *braille learning* (*N* = 2), and *safety/emergency preparedness* (*N* = 2, including 2 dual-coded). Table 2 presents BLV-specific apps organized by subcategory, with brief descriptions of common functions and features alongside examples of selected apps within each subcategory. Figure 4 visualizes the distribution of BLV-specific apps across subcategories.

**Table 2:** Apps targeting blind and low-vision populations

Category (N)	Description	App Examples
Environmental Accessibility (N = 46)^a^	Object detection and spoken, sound, or haptic feedback for environmental navigation	Blind Radar, iWalkStraight
	Provides information about local environment and points of interest for navigation	BlindSquare, FAR Vision Pro, Lazarillo - Accessible GPS
	Converts photos into audio descriptions of objects	Vision Assist, Envision AI
	Records surroundings immediately upon launching	Blindfold Video
	Separates speech from background noise	HeardThat
	Uses virtual reality to improve image clarity	iRis - Vision Through VR
	Alerts users with sound and vibration when Step-Hear devices are nearby	Step-Hear
	Helps users navigate transit by scanning tags from a distance or while moving	NaviLens
	Uses virtual reality glasses to support low vision tasks	SMW Patch
	Reads prescription medication labels aloud with visual assistance	ScripTalk Mobile, ScriptView Mobile
	Supports sailing navigation with GPS and haptic feedback	Seeing Assistant Sailing
	Uses sound cues to help users identify colors	Trace See
	Transcribes the National Park Service site paper brochures into acoustic media	UniDescription
	Identifies tactile paving and railroad tracks	Braille Block
	Magnifies objects in the environment (e.g., text on pill bottles, menus, makeup)	Big Cam Magnifier & Vision Aid
Provider Tools (N = 14)	Assesses eye discomfort and sensitivity with questionnaires and tests	CornealNeuropathicAssessment, Sensitivity Tests
	Patients compare cataract surgery lens outcomes	SMART Educator IOL Guide
	Tests for stereopsis using random-dot patterns	StereoTAB Stereopsis Testing
	Calculates Toric IOL values for eyecare providers	Biotech Calculators
	Explains lens functions and vision options to patients	Any I Consultant, MyReaderNumber
	Helps providers explain blue-coated lenses, blue light, and UV benefits	Bluespec HEV
	Supports tracking and management of binocular vision conditions	Bynocs
	Uses 3D imaging and narrated videos to explain eye diseases	Sight Selector Subscription
	Helps prescribe tilted prisms for diplopia	Tilted Prism
	Assesses near vision	Visucat N
Therapeutic / Rehabilitation (N = 14)	Provides exercises for children with cortical visual impairment	CViConnect, EDA PLAY TOM
	Connects low-vision users to rehabilitation	Alphapointe
	Utilizes eye movements, memory, and colored stimuli to exercise the brain	Brain Tool Kit, Find the Same
	Offers vision therapy games for amblyopia and related conditions	AmblyoPlay
	Supports training and progress tracking for low-vision users	DREX
	Improves navigation skills by training users to detect non-visual cues	Farmer Noah
	Therapeutic game that uses Gabor Patches to act on and improve the visual cortex area of the brain	GaborPatchGame, Gabor Brain Game, Sightseeing
	Recovery modalities for individuals with vision issues from neurological impairment	King Devick Recovery Practice
	Sight recovery app that uses 3D stereograms	Sight Recover 3D
	Builds visual motor skills using a digital light box	Visual LightPad
Digital Accessibility (N = 12)^a^	Provides digital image descriptions through human and artificial intelligence	From Your Eyes
	Large-print and high-contrast calculator app	Big Calculator Low Vision
	Magnifies text, photos, or videos on device	HumanWare explore Magnifier
	Mobile braille keyboard	MBraille
	Accessible news for blind/visually impaired	NFB Newsline Media
	Provides access to YouTube videos with an extra soundtrack to tell the viewer about text, illustrations, gestures, facial expressions, etc.	YouDescribeX
	Offers magnifier tools through AR, VR, or smart glasses	Digital Glasses for Low Vision, SuperVision for cardboard,
	Large font app for accessible notetaking	Big List
Education (N = 12)	Delivers blindness resources and event updates through notifications	GCB Link
	Teaches parents how to support children with blindness or low vision	4to24
	Newsletter for The Foundation Fighting Blindness	In Focus Newsletter
	Community radio stations, audio narrative newsletters, and podcasts	Sero
	Offers educational content on vision and eye disease	My Myopia Management, The Glaucoma Community
	Supports literacy development for children with CVI3	Bubbly CVI Literacy
Games (N = 7)	Audio-only game that utilizes hearing	Blinded
	Juggling game for low sighted individuals	Blindfold Juggle
	Audio game to develop hearing senses and spatial orientation	EchoVis Street, EchoVis Game
	Helps children develop visual and perceptual skills through a game	Eye Hero
	Audio gaming that uses vibrations to signal borders and movement	Tic Tac Toe by UNAR Labs
	3D game that supports visual recovery	Visual recovery 3D Konpeito
Vision Screening (N = 3)	Early screening of macular dysfunction	MaculaTester
	Provides at-home Amsler Grid tests for central vision loss	Amsler 3D, Amsler Grid App
Fitness (N = 3)	User receives real-time feedback about their fitness form using a video device, microphone, and Raspberry Pi to analyze exercise movements	BlindVision Fitness
	Offers audio-guided workouts and exercise instruction	ReVision Fitness
	Provides accessible workouts for users with physical or visual impairments	Accessercise
Braille Learning (N = 2)	Teaches Unified English Braille through lessons and challenges	Braille Academy: Play & Learn
	Comprehensive UEB learning with a live chat room, translators, and skill-application	Braille Decoded
Safety / Emergency (N = 2)^a^	Gives first responders quick access to patient special-needs information	AdaptCare
	Allows blind users to call emergency services and share their location	Sosfy Help

a Indicates apps are included in both DHH and BLV relevant subcategories (4 in environmental accessibility, 2 in digital accessibility, 2 in safety/emergency preparedness).

#### 3.3.1 Environmental accessibility

Environmental accessibility apps (*N* = 46) included features supporting BLV users’ navigation and interpretation of the physical environment. Navigation and orientation support included haptic and auditory feedback for real-time navigation, spoken information about nearby places or surroundings, identification of tactile paving and railroad tracks, and supports such as vibration or sound alerts when walking past audio beacons placed in public locations. Camera and vision-assist tools for interpreting the environment included object detection, camera-based spoken scene description, virtual reality (VR) to clarify captured images, object magnification, and glasses to color-correct and clarify images for individuals with conditions that affect central vision. Assistance with access to printed or labeled information in daily life included text-to-speech supports for reading prescription medication labels. Other apps included assistive technology that teaches low-vision individuals to understand images more clearly by associating colors with certain sounds using the C-major scale, and specialized or context-specific tools such as GPS and haptic-guided navigation assistance for sailing, National Park Service brochures made accessible in audio format, and rapid-start video recording features intended to support personal safety.

#### 3.3.2 Provider tools

Provider tool apps (*N* = 14) were designed to support vision specialists and clinics providing patient eye care via mobile applications, spanning screening and clinical assessment, patient education, and workflow support. Assessment and screening tools included questionnaires, tests, visual scales for eye discomfort and sensitivity, dot tests that measure depth perception, near-vision tests, intraocular lens calculators, and tools to help providers determine prism lens prescriptions. Patient counseling tools included educational resources covering eyeglass lens functions, blue light and UV protection, binocular vision conditions, and demonstrations of eye diseases using 3D images, narrated videos, and image-based scenario comparisons to support discussion of options for cataract surgery. Workflow and prescription support included patient session management and progress tracking.

#### 3.3.3 Therapeutic or rehabilitation

Therapeutic/rehabilitation apps (*N* = 14) offered tools to assist BLV users in rehabilitation, recovery, or ongoing management related to eye health. Condition-specific vision therapy and rehabilitation exercises included assessment or training support for individuals with partial vision loss, guided activities for children with cerebral vision impairment (CVI) emphasizing visual attention, focus, and related stimulation, and game-based therapy for amblyopia (lazy eye), strabismus (eye misalignment), and convergence insufficiency. Neuro-visual stimulation and perception training tools included activities using eye movements, memory tasks, color-based stimuli, striped visual patterns such as Gabor patches, 3D stereograms, and digital light-box activities to support visual processing and visual-motor skills. Mobility and orientation support included tools to help users detect non-visual environmental cues, build mental maps of their surroundings, and improve independent navigation. Service-related and recovery-focused tools connected users to eye rehabilitation services or provided support for vision challenges associated with neurological conditions.

#### 3.3.4 Education

Education apps (*N* = 12) provided informational resources related to BLV services, rehabilitation, and community support. Service navigation and organizational resources included blindness resource lists, contact information, and direct-call features to organizations such as the Georgia Council for the Blind. Community features included push notifications announcing programs and events. Family and caregiver-facing educational content included parent education modules for families of BLV children covering social skills, literacy, technology, academics, and daily living skills. Accessible educational media apps included audio-narrated newsletters, podcasts, community radio streams, and educational programming on general vision topics and eye-specific diseases. A subset of apps incorporated BLV child-oriented learning support, such as bright colors and engaging sounds to reinforce literacy-related skills.

#### 3.3.5 Digital accessibility

Digital accessibility apps (*N* = 12) promoted and assisted BLV users’ access to and interaction with digital content on technology devices. Accessible non-visual description and interpretation tools included image uploads for audio description, accessible news media, and YouTube video viewing tools that provide audio descriptions of on-screen text, settings, illustrations, gestures, and facial expressions. Visual enhancement and readability support included magnification of text, photos, or videos on mobile devices, a high-contrast calculator with large font sizes, and large font features for accessible note taking. Accessible input and productivity tools included mobile braille keyboards. Specialized assistive integrations included augmented reality (AR) or virtual reality (VR) headsets and digital magnifiers.

#### 3.3.6 Games

Game apps (*N* = 7) provided opportunities for BLV users to practice perceptual skills in engaging and interactive ways. Many games relied on auditory perception, using spatialized audio cues to guide users as they navigated audio-only virtual environments. For example, some simulations required users to rely solely on traffic sounds to safely cross a virtual street. In addition to skill-building, several games were primarily entertainment-focused, using audio or tactile feedback. For example, an app taught users to juggle through audio cues, while another enabled BLV users to play tic-tac-toe using vibration patterns.

#### 3.3.7 Vision screening, fitness, braille learning, and safety/emergency

Four categories contained three or fewer apps. Vision screening apps (*N* = 3) offered at-home screening tools to test for macular dysfunction and other conditions affecting central vision. These apps typically used interactive and audio-guided forms of the Amsler Grid, a commonly used clinical screening tool. Fitness apps (*N* = 3) were designed to support safe participation in exercise and physical activity for BLV users. Braille learning apps (*N* = 2) provided accessible platforms for users to learn, practice, and test their knowledge of Unified English Braille. Features included structured lessons, short stories for reading practice, and live chatrooms. For example, one lesson format included 59 levels, 29 challenges, and 13 instructional sections, with certificates awarded upon completion. Content commonly covered letters, numbers, punctuation, special symbols, word signs, group signs, and contractions. Safety/Emergency apps (*N* = 2) offered accessible ways for users to contact emergency services and communicate key information quickly. Common features included geolocation tools that share a user’s location with emergency responders and interfaces that allow users to make phone calls. One app used QR codes linked to a user profile that responders can scan to access a customized care plan and relevant support needs.

### 3.4 Fitness and nutrition-specific apps

Across a total of 1,735 unique retrieved apps, 379 (21.8%) were categorized as targeting DHH and/or BLV populations. However, only four met criteria for DHH- or BLV-tailored fitness apps, representing 0.23% of retrieved unique apps. These were BW Dance, ReVision Fitness, BlindVision Fitness, and Accessercise. No DHH- or BLV-tailored nutrition apps were identified. The four tailored fitness apps were compared using the same domains: developer name, direct and indirect costs, selected fitness offerings, and analyzed accessibility features, as presented in Table 3.

**Table 3:** Fitness app accessibility.

	BlindVision Fitness	ReVision Fitness	BW Dance	Accessercise
Developer	Coding Minds, Inc.	ReVision Training LLC	Interactive Group FCZ	Accessercise LTD
Costs				
Direct	Free	14-day free trial	Free	Free version with limited features (7-day free trial)
		$5.99 monthly		$13.99 monthly
		$59.99 annually		$34.99 for 3 months
				$55.99 for 6 months
				$75.99 for 1 year
Indirect	Raspberry Pi, Microphone, Camera, Tripod (∼$200)	N/A	Cost of downloading music	N/A
Fitness Offerings				
Online workouts	No	Yes	No	Yes
Live workouts	No	No	No	No
Fitness tracking	No	Yes	No	Yes
Goal setting	No	Yes	No	Yes
Networking / interaction	No	Yes	No	Yes
Reminders / scheduling	No	No	No	Yes
Other	Verbal feedback for correct exercise form	Classroom, fitness studio, fitness plans	Freestyle (non-instruction) dancing	Customizable searches
BLV Accessibility				
Text (size, contrast)	No	Yes	Yes	No
Magnification / zoom	No	No	No	No
Non-visual workout guidance	Yes	Yes	No	Yes
Haptics / tactile cues	No	No	Yes	No
Navigation / touch targets (large buttons)	No	Yes	No	Yes
Voice commands / control	Yes, through Raspberry Pi	No	No	No
DHH Accessibility				
Captions / subtitles	No	No	No	Yes
Searchable transcripts for workouts / classes	No	Yes	No	No
Visual / haptic alerts	No	No	Yes	No
Audio-independent instructions	No	Yes	No	Yes
Alerts and notifications	No	No	No	Yes

#### 3.4.1 BW dance

BW Dance is designed for deaf and hard-of-hearing individuals. The app is designed to create a freestyle, non-instructional dance environment supported by haptics and visual cues. The app enables users to sense the rhythm of music by converting audio into flashing lights and vibration patterns. Users may choose a play mode (vibration only, flash only, or vibration paired with flash). Individual songs or albums must be purchased if users desire to include music, since streaming services are not supported. BW Dance appeared in only one of the nine searches, the *Deaf health fitness* search in the general store, as the 161st app returned out of 176 results.

#### 3.4.2 ReVision fitness

ReVision Fitness includes a Classroom section, a Fitness Studio, and Fitness Plans. Full audio descriptions are available throughout the app. The Classroom section includes structured instructional lessons designed by a blind personal trainer and Paralympic athlete tailored to BLV individuals. The purpose of each exercise, proper form, common mistakes, and possible modifications is explained. The Fitness Studio contains preset audio-guided workouts, and Fitness Plans provide goal-oriented programming. Additional features include the My Stats page, where users can input age, sex, height, current weight, goal weight, average activity level, experience level, and goals. Based on user input data, the My Stats page displays the user’s Body Mass Index (BMI), BMI category, ideal body weight range, basal metabolic rate (BMR), caloric consumption rate, recommended calorie intake, weight change, and time to goal weight. Users can also participate in four basic fitness tests to assess general strength and endurance, and their results can be benchmarked against others of their same age and sex. Other notable features include a Recommended Products section with curated accessible equipment, a collection of Health and Fitness Guides offering in-depth explanations of various fitness topics, and a detailed frequently asked questions (FAQ) section. ReVision Fitness included online workouts, fitness tracking, goal setting, and networking or interaction features, as well as accessible text and contrast features, non-visual workout guidance, searchable transcripts for workouts or classes, and audio-independent instructions. The app offers a 14-day free trial, after which continued use requires a paid subscription of $5.99 per month or $59.99 per year. ReVision Fitness appeared in four searches. In the general store *blind health fitness* search, it appeared as the 8th app out of 171. However, when searching *blind* in the marketplace’s Health & Fitness category, it appeared as the 130th app out of 158. It also appeared in the general *hard of hearing health fitness* search, as the 101st app out of 180. In the general *hearing loss health fitness* search, it appeared as the 126th app out of 170.

#### 3.4.3 BlindVision fitness

BlindVision Fitness uses a Raspberry Pi–powered microphone and camera system to analyze user movement through AI-based pose detection. The external hardware provides real-time voice feedback on form and technique, designed to assist BLV users in completing exercises safely and accurately. Non-visual workout guidance is offered for seven specific exercises in the form of verbal feedback on correct exercise form. The seven exercises included bench press, bicep curl, deadlift, downward dog, plank, pushups, and squats. BlindVision Fitness did not include online workouts, tracking, goal setting, networking, or reminders. The app depends on external equipment that may be costly and difficult to set up, even for sighted users, potentially limiting usability. BlindVision Fitness appeared in two searches. In the general *blind health fitness* search, it appeared as the 5th app returned out of 171 results. In the Health & Fitness category of the App Store, when using the keyword *blind*, it appeared as the 10th app returned out of 158 results.

#### 3.4.4 Accessercise

Accessercise is a disability-tailored fitness app that provides structured exercise content for individuals with a wide range of impairments, including upper or lower limb amputation, spinal cord injury, dwarfism, and partial or full visual impairment. Key features include structured workout programs, exercises demonstrated by people with disabilities, and workouts that can be done in a variety of settings such as at home, outdoors, or in a gym. Users can filter workouts by setting to match personal preferences and available equipment. For users with partial or full visual impairment, the app includes step-by-step verbal explanations of each exercise to support accessibility and safety. Accessercise included online workouts, fitness tracking, goal setting, networking or interaction, and reminders or scheduling, as well as non-visual workout guidance, large navigation or touch targets, captions or subtitles, and audio-independent instructions. Users can search for nearby gyms; however, this feature was limited to the United Kingdom. Accessercise offers a 7-day free trial, after which continued use requires a paid subscription of $13.99 per month or $75.99 per year. Accessercise appeared in two of the nine searches. When searching *deafblind* in the Health & Fitness category of the App Store, it appeared as the 96th app out of 176 results. When searching *blind health fitness* in the general store, it appeared as ​the 161st app returned out of 171 results.

#### 3.4.5 Comparison of fitness apps

The four apps varied considerably in terms of fitness offerings and accessibility support. ReVision Fitness and Accessercise offered a broader set of fitness functions, including online workouts, fitness tracking, goal setting, and networking or interaction features. Accessercise included reminders and scheduling functions. BlindVision Fitness was narrower but distinctive in offering real-time verbal form feedback through external hardware. BW Dance provided haptic and visual rhythm support for freestyle dance. Across apps, no live workout features were identified.

Accessibility features varied across apps. Non-visual workout guidance was present in the three BLV-relevant fitness apps: BlindVision Fitness, ReVision Fitness, and Accessercise. DHH-relevant accessibility features were less consistent. Accessible text size/contrast features were found in two apps: ReVision Fitness and BW Dance. Navigation/touch targets were found in two apps: ReVision Fitness and Accessercise. Voice commands/control was found in one app, BlindVision Fitness, through use of a Raspberry Pi. Researchers could not easily locate magnification or zoom features across the four fitness apps. ReVision Fitness and Accessercise included audio-independent instructions. Searchable transcripts for workouts or classes were found for ReVision Fitness. Accessercise included subtitles and alerts/notifications.

Cost and access barriers included ongoing subscriptions for the full versions after a short trial period, expensive external hardware requirements for BlindVision Fitness, and optional external music purchase for BW Dance. Search discoverability also varied, with some apps appearing near the top of relevant searches and others appearing far lower in the results.

## 4 Discussion

### 4.1 Discoverability barriers

A central finding of this study is that DHH/BLV-tailored fitness apps were rare and difficult to locate in Apple App Store search results. Across the final corpus of 1,735 unique apps, disability-related keyword searches produced substantial noise. Nearly 60% of apps returned in the searches were either general-audience health apps or unrelated to health and fitness. Only a minority of apps were explicitly relevant to DHH or BLV users, and only four apps were identified as DHH/BLV-tailored fitness apps. No DHH/BLV-tailored nutrition apps were found.

A key contribution of this work is its focus on search returns as a proxy for real-world access. From a user perspective, the App Store is not merely an index of available tools; it is a ranked, algorithmically mediated interface that shapes what users can efficiently locate (26). Discoverability depends on multiple factors, such as app store product categorization, rankings, ratings and reviews (27), search algorithms (28), search specificity (29), user engagement and metadata, including titles, keywords, and descriptions (30, 31), visual assets, such as fonts, styles, logos, and colors (32), feature discoverability (33), and clarity about accessibility features and functions (32).

The high proportion of general or unrelated apps in the final corpus suggests that DHH/BLV users may need to expend substantial effort to identify truly tailored offerings, particularly if they rely on common identity terms (e.g., Deaf, blind) and the default search interface. This challenge is compounded by the finding that many hearing and vision-related apps are not disability-targeted. For example, general hearing subcategories in the corpus included sign language learning apps intended for the general public, tinnitus relief apps, and general hearing tests and screenings. Similarly, general vision subcategories included at-home vision tests and screenings, generic eye health apps, and simulation apps intended for sighted users. These patterns indicate that disability-related keywords often retrieved apps associated with hearing or vision, but not necessarily apps designed for DHH/BLV users or their accessibility needs.

The implications for public health impact are substantial. Even well-designed accessible apps cannot benefit intended users if they are difficult to find, poorly described in metadata, or buried within search results. From this perspective, discoverability may function as a structural determinant of access similar to transportation barriers or inaccessible physical infrastructure, because it affects whether DHH/BLV users can locate tools that support health behaviors, healthcare engagement, and safer participation in daily activities. Only five search terms yielded a relevant fitness result at all: *Deaf, blind, hard of hearing, hearing loss,* and *deafblind*. Notably, the *hard of hearing* and *hearing loss* searches yielded results for a BLV-targeted app rather than a DHH-targeted app. Search success was inconsistent across the general store and the Health & Fitness category. For example, some relevant apps appeared relatively early in search rankings, such as ReVision Fitness in the general *blind health fitness* search and BlindVision Fitness in both the general *blind health fitness* search and the Health & Fitness *blind* search. In contrast, BW Dance and Accessercise appeared far later in search rankings, including at the 161st and 96th positions, respectively, making them unlikely to be found through typical searching behavior. These patterns suggest that access depends on more than whether a relevant app exists. Access also depends on where the app appears, which terms index it, and whether users search in the general store or within a category filter. Future work should investigate the user experience of marketplace searches more directly, including time-to-find metrics, click behavior, and the search strategies most likely to surface accessible tools.

### 4.2 Disability-tailored health and fitness in a mainstream marketplace

Although the initial aim of the study was to identify disability-targeted fitness and nutrition apps, the results indicate that the health and fitness ecosystem available to DHH/BLV users in the App Store is largely composed of digital and environmental navigation tools rather than fitness or nutrition programming. This study identified a diverse and innovative ecosystem of mobile tools relevant to DHH and BLV users, particularly in the areas of communication access, environmental navigation, device support, rehabilitation, education, and safety. These findings demonstrate the creativity and breadth of current mobile health innovation for users with disabilities. At the same time, the study revealed a pronounced gap in disability-tailored fitness and nutrition tools and major barriers to locating them in a mainstream marketplace. For many DHH/BLV individuals, participation in exercise, healthy eating habits, and other health behaviors depends on foundational accessibility support that makes physical activity and meal preparation feasible and safe. In this way, exercise and nutrition behaviors may be supported by apps that assist with communication access, environmental navigation, device support, rehabilitation, and safety.

#### 4.2.1 DHH health and fitness

For DHH users, the app ecosystem was dominated by hearing aid-related apps. The prominence of this category suggests that financial factors may drive the substantial share of marketplace offerings for DHH users. This concentration likely reflects commercial incentives for proprietary products, where manufacturers can deliver utility and support for hearing aids while increasing long-term user engagement through device-linked features. At the same time, the dominance of hearing-aid apps demonstrates how mainstream marketplaces often frame DHH users primarily through a medicalized hearing loss lens, particularly oriented toward late-deafened or older adult users, rather than through the needs and preferences of profoundly or culturally Deaf individuals who primarily communicate through signed languages and seek tools centered on communication access rather than hearing restoration (34).

Apart from hearing aid-related apps, a variety of apps assisted DHH users with environmental and digital communication access, including sound amplifiers, live captioning, video interpretation, and alert tools using haptics. Other apps related to emergency preparedness, safety, and community connection. These categories align with practical needs that support health engagement, including the ability to communicate with providers and emergency personnel, access health-related information, safely navigate situations in which auditory cues are typically relied on, and network with other community members who may support mental well-being. Compared with the breadth of general-audience fitness offerings in the marketplace, there appears to be limited availability of DHH-targeted technologies that directly support health behaviors such as physical activity and nutrition, suggesting that marketplace innovation for DHH users is currently weighted toward device management and accessibility tools rather than health-behavior change and inclusive fitness and nutrition programming.

#### 4.2.2 BLV health and fitness

For BLV users, environmental accessibility apps were the most common category. These tools frequently supported navigation and orientation through haptic and auditory feedback, spoken information about nearby places, identification of tactile paving or hazards, and transit-related supports. A complementary cluster focused on camera- and vision-assist functions, including object detection, spoken scene description, image clarification, and magnification. Other apps supported access to health activities through text-to-speech for prescription drug labels, safety functions such as rapid-start recording, and specialized contexts such as GPS and haptic-guided navigation for recreational activities. Games and braille learning apps further supported skill development and accessibility for everyday life. In aggregate, these features demonstrate the role of digital health tools as portable sensory platforms for environmental navigation and interpretation, which are an essential foundation for independent mobility and daily safety, including participation in health-related activities. The BLV ecosystem also included provider tools and therapeutic/rehabilitation apps, reflecting a clinical orientation in vision-related app development for users with a variety of visual conditions. While these tools may support mobility, safety, and clinical care, few directly addressed accessible fitness participation or sustained health behavior change.

### 4.3 Accessibility features of targeted fitness apps

One of the most striking findings was the scarcity of DHH- and BLV-targeted fitness and nutrition apps in the App Store search returns. Among DHH-specific apps, only one fitness app was identified. Among BLV-specific apps, only three fitness apps were found. No DHH- or BLV-specific nutrition apps were identified. This finding suggests that disability-tailored fitness and nutrition tools are either underdeveloped, poorly marketed or indexed, or not made visible through common App Store searches.

The small subset of DHH- and BLV-focused fitness apps offered a relatively limited range of features compared with popular mainstream fitness apps. Among the four analyzed apps, non-visual workout guidance was the most frequent feature found for BLV users, while the DHH-focused app centered on freestyle dance through visual and haptic rhythm cues. Real-time feedback on exercise form for BLV users was available for BlindVision Fitness using a Raspberry Pi. However, only two of the four apps offered online workouts, fitness tracking, goal setting, or networking features. Only one app offered captions, scheduling tools, and notification features. Across the four apps, no live workouts or magnification/zoom features were easily located. These patterns suggest that although the identified apps incorporated certain meaningful accessibility features, some lacked workout content variety, tracking and planning tools, positive reinforcement, and social engagement options present in many popular mainstream fitness apps that motivate sustained long-term engagement (35).

Several factors may contribute to the scarcity and limited breadth of existing tailored fitness apps. Developing accessible fitness content can be technically and resource intensive, requiring high-quality captions and transcripts (36), audio-independent or visual-independent instruction (36), carefully designed visuals, haptics, or additional tools such as larger element size, custom contrast, color adjustments, magnification software, auditory alerts (37), non-visual navigation and feedback (38), and product development that includes robust testing with users with disabilities (39). Market incentives may favor device-linked companion apps for hearing aids, or broadly marketable accessibility utilities rather than niche fitness or nutrition programming. Finally, some developers may assume that built-in OS accessibility features are sufficient, even though accessible interface features do not automatically translate into accessible fitness instruction, content delivery, or navigation design.

Cost also emerged as a potential barrier across the small number of tailored fitness apps identified. Although the barriers took different forms, all four apps involved direct or indirect costs that could limit use. In addition, some apps offered a limited exercise library or narrow range of activities, which may reduce long-term value for users relative to their financial or initial setup burden. Thus, the fitness-app gap includes both limited availability and potential affordability or other usability barriers.

### 4.4 Recommendations

Marketplace design, developer practices, and engagement of people with disabilities each play important roles in improving the discoverability and usefulness of tailored fitness apps. App stores are internally structured according to classification systems that label each app according to one or more classes to which it belongs (40). This organizational framework is intended to facilitate user navigation and improve the efficiency of locating relevant apps. However, the present findings suggest that category assignment alone may be insufficient for helping DHH/BLV users locate relevant tools. Since App Store searches for fitness apps targeting BLV and DHH users produced a large proportion of unrelated or general-audience apps, users may have difficulty finding tools designed for their needs.

App marketplaces could improve discoverability through more specific accessibility metadata and filtering options. Practical changes could include disability-specific tags, such as *Deaf/hard-of-hearing* or *Blind/low-vision*, as well as feature-specific labels for captions, sign-language content, audio description, screen-reader compatibility, haptic cues, large text, high contrast, non-visual workout guidance, and audio-independent instruction. These labels could be incorporated into search filters, app listing pages, and ranking systems so that accessibility features are easier to identify when users search. Curated collections of accessible health and fitness apps, developed in partnership with disability organizations, rehabilitation specialists, and DHH/BLV users, may also improve the visibility of relevant tools.

Study results additionally highlight opportunities for developers to improve discoverability. Developers classify and describe their apps in the marketplace, and clearer accessibility metadata describing the accessibility characteristics of the app may improve discoverability (41). Developers should use plain-language descriptions of target users and accessibility features and include relevant community-preferred keywords in app titles, descriptions, and keywords. Usability guidelines emphasize that systems should make available features immediately recognizable and easy to understand (42), with aesthetics and design features that enhance user appeal (43). For fitness and nutrition apps, this means that accessibility features should not be hidden within general marketing language or screenshots. Instead, accessibility features should be explicitly described.

General-audience fitness apps should consider adapting their interface design and instructional content to be accessible for DHH and BLV users. In addition, accessible fitness apps should deliver instruction and feedback through multiple sensory channels and adopt more inclusive design practices. For example, DHH users may benefit from captioning, signed instruction, clear visual demonstrations, and haptic prompts, while BLV users may benefit from structured audio guidance, voice navigation, high-contrast interfaces, large touch targets, and real-time auditory feedback on movement or form. Attention to cost barriers is also important, as subscription requirements and specialized hardware or separate media purchases may limit adoption for some users.

Finally, engaging disability communities throughout the design process is essential for developing usable and relevant technologies. Developers without lived experience with hearing or visual disabilities may not recognize important needs related to usability. For instance, users with visual impairments report that buttons in digital health tools should be clearly differentiated in shape, color, and size, while users with hearing aids or cochlear implants have noted that high-pitched default voices may create listening barriers (39). Participatory design approaches that involve DHH and BLV individuals can help ensure that apps reflect real-world needs and preferences. Usability testing with disabled users may also help refine accessibility features, clarify instructions, identify navigation challenges, and improve adoption. Partnerships with disability organizations and rehabilitation specialists may further support DHH/BLV users in finding these resources through trusted community networks, ultimately improving the relevance and adoption of accessible health and fitness technologies. Given the scarcity of disability-tailored fitness and nutrition apps identified in this study, future work should also evaluate the accessibility of mainstream fitness and nutrition apps for DHH and BLV users. General-audience platforms may already contain features that could be adapted through user-centered redesign and systematic testing with DHH and BLV participants.

### 4.5 Limitations

This study has several limitations. First, only the Apple App Store marketplace in the United States was searched, limiting generalizability. Results may not reflect apps available in other countries, languages other than English, or other marketplaces such as Google Play, which differ from the Apple App Store in developer policies, metadata fields, ranking algorithms, user base, device diversity, and app availability. Therefore, the specific apps, ranking positions, and discoverability patterns observed should be interpreted as results from one marketplace context rather than a comprehensive global assessment of DHH/BLV health and fitness apps. Future studies should compare discoverability across the Apple App Store, Google Play, and other app ecosystems.

Second, because app store inventories are dynamic, the corpus represents a time-bound snapshot of search returns collected between October 2025 and February 2026. Search rankings may vary by time, geographic location, language settings, device type, operating system version, user history, app popularity, paid promotion, and changing algorithms. Apps may change their names and descriptions, and app availability may shift over time, limiting reproducibility of the observed app ecosystem. For example, some apps identified during Phase 1 were no longer available by Phase 3, and others may have been newly released, removed, or re-labeled after data collection. Although personalization was minimized by using factory-reset iPads running the same iPadOS version, the study could not eliminate all algorithmic or contextual influences on search results. The ranking positions reported here should therefore be interpreted as discoverability at the time of the search rather than a reproducible fixed ordering of apps. Repeating the same searches may yield different apps, ranking positions, or levels of discoverability. Future research should repeat searches across multiple time points, devices, regions, and language settings to evaluate the stability of app rankings and the robustness of DHH/BLV app discoverability.

Third, the search strategy relied on predetermined keywords and the App Store’s search algorithm. Relevant apps may not have been captured if they used less obvious disability-related terminology or were poorly indexed within App Store categories. Although the strategy intentionally included both community-preferred and commonly used mainstream disability terms, some potentially relevant apps may still have been missed.

Finally, app classification and feature/function synthesis depended primarily on developer-provided listing descriptions and screenshots. These descriptions may not fully reflect actual usability, accessibility, feature quality, or user experience. Although fitness-focused apps were downloaded and tested to observe functions and features, most apps were not directly evaluated for usability, accessibility, quality, effectiveness, clinical accuracy, or privacy. Additionally, the accessibility assessment of the fitness apps should be interpreted as a brief evaluation of available fitness activities and easily observable accessibility features rather than actual usability among DHH/BLV users. WCAG-informed review cannot fully capture all user needs, preferences, or interaction barriers. Since no DHH or BLV participants were included in the app testing phase, the assessment may not reflect lived user experience, instruction comprehension, satisfaction, or sustained engagement. Future work should combine guideline-based assessment with human evaluation, including usability testing and participatory evaluation with DHH and BLV users.

Given the lack of available fitness apps targeting DHH and BLV users, future studies should also evaluate the accessibility of mainstream fitness apps for these populations. Additional research should also examine how ranking position, category filtering, and app metadata shape real-world search success for users with disabilities, including which combinations of search terms are most likely to surface relevant tools.

### 4.6 Conclusion

Results from this scoping App Store search, content analysis, and accessibility assessment suggest that DHH/BLV users searching a mainstream marketplace for health and fitness tools are likely to encounter a landscape dominated by general-audience apps and adjacent hearing- or vision-related apps that are not disability-targeted. Among 1,735 unique apps, most search returns were general health/fitness or unrelated, and only 379 apps were relevant to DHH and/or BLV users. Within that subset, many apps primarily supported hearing aid management, communication access, environmental navigation, digital accessibility, or rehabilitation rather than direct fitness programming. Only four apps were identified as DHH- or BLV-tailored fitness apps, and no DHH/BLV-tailored nutrition apps were found. These findings suggest that the current app ecosystem offers more access-oriented support than direct disability-tailored fitness content.

The limited number of disability-tailored fitness apps, combined with barriers related to app discoverability, highlights a persistent gap between the potential of mobile health technologies to improve health and fitness equity and their practical benefits for DHH/BLV communities. Improving discoverability, variety of offerings, and affordability is essential if mobile health technologies are to better support fitness equity for DHH and BLV users. Marketplace design, including clearer metadata, filtering, and tags, developer practices that make accessibility features easier to identify, and participatory testing with people with disabilities, are important steps toward closing this gap.

## Data Availability

The original contributions presented in the study are included in the article/Supplementary Material, further inquiries can be directed to the corresponding author.
