# Supplementary material for: Discoverability and characteristics of health and fitness apps for deaf/hard-of-hearing and blind/low-vision users
Source: Front Digit Health. 2026 Aug 17;8:1864883. doi: 10.3389/fdgth.2026.1864883 (PMC13526966; doi:10.3389/fdgth.2026.1864883)
Supplement: Supplementary file 1 [file Datasheet1.pdf]

Supplementary Material: Codebook Categories and Descriptions

BLV Categories

1. **BLV Digital Accessibility:** Assists in using the device to enable or improve access to content and tasks on the device itself for BLV users
2. **BLV Environmental Accessibility:** Helps BLV users access, interact with, or navigate the environment outside the device.
3. **BLV Education:** Provides informational resources related to BLV services, rehabilitation, community support, etc.
4. **BLV Fitness:** Directly supports fitness activities for BLV users.
5. **BLV Games:** Provides opportunities for BLV users to practice perceptual skills or provides entertainment in gamified interactive ways. Note: games that were primarily therapeutic in nature should be categorized in the therapeutic/rehabilitation category.
6. **BLV Provider Tools:** Designed to support vision specialists and clinicians providing patient eye care for BLV users, spanning clinical assessment, education for providers, and treatment planning/decision support.
7. **BLV Therapeutic/Rehabilitation:** Offers modalities to assist BLV users in healing, recovery, or ongoing management of eye health.
8. **BLV Vision Screening:** Offers at-home screening tools to test for macular dysfunction and other vision disturbances.
9. **BLV Braille Learning:** Platforms for users to learn, practice, and test their knowledge of Braille.
10. **BLV Safety/Emergency Preparedness:** Provides accessible ways for users to contact emergency services and communicate key information quickly.

DHH Categories

11. **DHH Digital Accessibility:** Assists in using the device to enable or improve access to content and tasks on the device itself.
12. **DHH Environmental Accessibility:** Helps DHH users access, interact with, or navigate the environment outside the device.
13. **DHH Clinical Home Monitoring:** Enables patients to communicate medical results, condition monitoring information, or maintain an active and ongoing relationship with their provider.
14. **DHH Community/Religion:** Supports social connection and community engagement with other DHH community members, or between hearing individuals (e.g., parents of DHH children) and Deaf community members.
15. **DHH Education:** provides informational and instructional resources related to issues affecting DHH individuals, hearing loss, communication strategies, or skill development.
16. **DHH Fitness:** Directly supports fitness activities for DHH users.
17. **DHH Fitness Club:** Fitness clubs (physical gathering outside of device) for fitness activities specifically for DHH individuals.
18. **DHH Provider Tools:** Designed to support healthcare professionals and specialists working with DHH patients.
19. **DHH Research:** Platforms to disseminate research relevant to DHH issues.
20. **DHH Safety/Emergency Preparedness:** Accessible ways for DHH users to contact emergency services and communicate key information quickly.
21. **DHH Sign Language Learning:** Tailored toward sign language learning and advancement for DHH users.
22. **DHH Therapeutic/Rehabilitation:** Provides rehabilitation exercises for individuals with cochlear implants, hearing aids, auditory processing disorders, aphasia, etc.
23. **Hearing Aid Related:** Designed to support the use, customization, and maintenance of hearing aid devices.
24. **Sound Amplifier:** Focused on enhancing auditory input from the user’s immediate environment.

DHH/BLV Categories

25. **DHH/BLV Digital Accessibility:** Assists in using the device to enable or improve access to content and tasks on the device itself, included features for both DHH and BLV users.
26. **DHH/BLV Environmental Accessibility:** Helps both DHH and BLV users access, interact with, or navigate the environment outside the device.
27. **DHH/BLV Safety/Emergency Preparedness:** Accessible ways for both DHH and BLV users to contact emergency services and communicate key information quickly.

General Health/Unrelated (not tailored to DHH/BLV users)

28. **General Health/Fitness**: Fitness apps created for use by the general public.
29. **Provider/Insurer Connection:** Tools to connect patients to providers, insurance companies, or retail stores
30. **Provider Tools:** Mobile tools to help providers serve patients.
31. **Other Disability:** Disability apps not related to DHH or BLV populations.
32. **Research:** Applications related to research but unrelated to BLV and DHH.
33. **Safety/Emergency Preparedness:** Accessible ways for users to contact emergency services and communicate key information quickly.
34. **Studio/Club:** Studio/club–specific or geographically restricted gym apps with a physical location.
35. **Other/Unrelated:** Apps unrelated to search terms that do not fit into categories above.

General Hearing (not tailored to DHH)

36. **Tinnitus:** Provides therapeutic relief for individuals experiencing tinnitus.
37. **Hearing Test/Screening:** Designed for the general public to assess hearing ability.
38. **Hearing Simulator:** Created for non-DHH individuals to experience what it is like to be deaf or hard-of-hearing.
39. **Sign Language Learning:** Apps that taught any type of sign language, catered to the general public, and were not intended specifically for DHH individuals.
40. **Generic Hearing Health:** Aimed to improve hearing, measure real-time noise levels, customize audiograms to personalize hearing profiles, etc.

General Vision (not tailored to BLV)

41. **Vision Tests/Screening:** Provides at-home examinations and tests for the general public to detect general vision abilities including visual acuity, color blindness, contrast clarity, etc.
42. **Generic Eye Health:** Generic eye health apps including eye exercises intended to maintain or improve eyesight, reminders for users to take screentime breaks and blink, visual stress diagnosis, etc.
43. **Vision Therapeutic/Rehabilitation:** Aims to treat or relieve general eye symptoms (not specific to BLV).
44. **Vision Simulator:** Offers BLV simulation experiences for sighted individuals.
45. **Colorblind Support:** Provides resources for colorblind individuals, including color recognition and perceptual distinctiveness functions. Note: Apps aiming to test for colorblindness should be categorized in vision tests/screening.
46. **Eye Disease Screening:** Apps created to detect various eye diseases (apart from general tests and screenings to assess visual acuity, colorblindness tests, contrast clarity, etc.)
47. **Vision Clinical Home Monitoring:** Apps that aid in monitoring general eye health and connecting users with an eye care professional.
